# Plan quality assessment of modern radiotherapy delivery techniques in left-sided breast cancer: an analysis stratified by target delineation guidelines

**DOI:** 10.1259/bjro.20200007

**Published:** 2020-12-11

**Authors:** Irfan Ahmad, Kundan Singh Chufal, Chandi Prasad Bhatt, Alexis Andrew Miller, Ram Bajpai, Akanksha Chhabra, Rahul Lal Chowdhary, Anjali Kakria Pahuja, Munish Gairola

**Affiliations:** 1 Department of Radiation Oncology, Rajiv Gandhi Cancer Institute and Research Centre, Sector 5, Rohini, New Delhi, India; 2 Department of Radiation Oncology, Sarvodaya Hospital and Research Centre, Sector 8, Faridabad, Haryana, India; 3 Department of Radiation Oncology, Illawara Cancer Care Centre, Wollongong NSW 2500, Australia; 4 School of Medicine, Keele University, Staffordshire, United Kingdom

## Abstract

**Objective:**

This study compares planning techniques stratified by consensus delineation guidelines in patients undergoing whole-breast radiotherapy based on an objective plan quality assessment scale.

**Methods:**

10 patients with left-sided breast cancer were randomly selected, and target delineation for intact breast was performed using Tangent (RTOG 0413), ESTRO, and RTOG guidelines. Consensus Plan Quality Metric (PQM) scoring was defined and communicated to the physicist before commencing treatment planning. Field-in-field IMRT (FiF), inverse IMRT (IMRT) and volumetric modulated arc therapy (VMAT) plans were created for each delineation. Statistical analyses utilised a two-way repeated measures analysis of variance, after applying a Bonferroni correction.

**Results:**

Total PQM score of plans for Tangent and ESTRO were comparable for FiF and IMRT techniques (FiF *vs* IMRT for Tangent, *p* = 0.637; FiF *vs* IMRT for ESTRO, *p* = 0.304), and were also significantly higher compared to VMAT. Total PQM score of plans for RTOG revealed that IMRT planning achieved a significantly higher score compared to both FiF and VMAT (IMRT *vs* FiF, *p* < 0.001; IMRT *vs* VMAT, *p* < 0.001).

**Conclusions:**

Total PQM scores were equivalent for FiF and IMRT for both Tangent and ESTRO delineations, whereas IMRT was best suited for RTOG delineation.

**Advances in knowledge:**

FiF and IMRT planning techniques are best suited for ESTRO or Tangent delineations. IMRT also yields better results with RTOG delineation.

## Introduction

Breast irradiation is essential in the management of breast cancer after breast-conserving surgery (BCS) and contemporary delivery techniques rely on consensus delineation guidelines to reduce long-term cardiac morbidity, especially in left-sided breast cancer.^1–4^

One of the most widely used delineation guideline is the RTOG 0413 WBI (Tangent) Protocol, which includes all clinically palpable breast tissue in its tangential design.^5^ The adoption of CT-based radiotherapy planning and the lack of an anatomical basis in this guideline drove the development of two consensus guidelines: the RTOG (Radiation Therapy Oncology Group) and the European SocieTy of Radiation Therapy (ESTRO) consensus guidelines.^3,4^

The RTOG consensus guideline provides anatomical bony and muscular landmarks for clinical target volume (CTV) delineation.^3,5^ In contrast, the ESTRO consensus guideline provides vessel-based landmarks to define the medial and lateral extent of the breast tissue, and recommends a ventral retraction of caudal CTV to distinguish abdominal fat from mammary fat.^4^ Both RTOG and ESTRO consensus guidelines also recommend adding a planning target volume (PTV) margin to the delineated CTV, in contrast to the RTOG 0413 WBI (Tangent) target which is delineated directly as a PTV.^3–5^

While the dosimetric performance of different WBI delivery techniques has been compared, these analyses have not contemporaneously addressed the issue of different delineation techniques. Our primary objective is to address this gap in the literature by investigating the performance of three treatment planning techniques [(Field-in-Field Intensity Modulated Radiotherapy Technique (FiF), inverse optimised Intensity Modulated Radiotherapy Technique (IMRT) and Volumetric Modulated Arc Therapy (VMAT)] for targets derived from the three different target delineation protocols (RTOG 0413, RTOG Consensus Guideline and ESTRO Consensus Guideline).

## Methods

Ten patients with left-sided breast cancer were selected using a random number generator from our institutional database. All had undergone BCS followed by adjuvant radiotherapy (46 Gy/23 Fx followed by an electron boost to lumpectomy cavity 12.5 Gy/5 Fx).

Patients underwent a free-breathing contrast-enhanced CT scan (Siemens Somatom Sensation Open; slice thickness 2 mm) on a carbon fibre breast board (Klarity Medical Products, USA) in the supine position and immobilised in a 4-point thermoplastic cast (Orfit Industries, Belgium). Wire markers were placed on the patient’s breast to mark the maximal palpable extent of the breast.

### Target volume and organs at risk (OAR) delineation

The institutional practice for the original delivered WBI treatment used a tangential planning target volume (PTV) delineated according to the RTOG 0413 WBI protocol without a CTV.^5^ The PTV was cropped at the lung-chest wall interface, and the lung depth did not exceed 3 cm. For this study, this volume was named PTV_Tang_Plan.

The simulation CT was retrieved and re-contoured according to the RTOG consensus and ESTRO consensus guidelines to produce CTV_RTOG and CTV_ESTRO, respectively.^3,4^ A 5 mm isotropic margin was added (limited by the skin, lung-chest wall interface and not allowed to cross-the midline) to create PTV_RTOG_Plan and PTV_ESTRO_Plan, respectively.

These three PTV’s were used for plan optimisation (detailed below) by Field-in-Field Intensity Modulated Radiotherapy Technique (FiF), inverse optimised Intensity Modulated Radiotherapy Technique (IMRT) and Volumetric Modulated Arc Therapy (VMAT).

For plan evaluation, the three PTV’s were copied to evaluation structures, PTV_Tang_Eval, PTV_RTOG_Eval, and PTV_ESTRO_Eval, respectively, after cropping 5 mm from the skin surface (the body contour auto-generated by the TPS with a threshold set at −350 HU). The skin is not anatomically a part of the breast except at the nipple, nor is it considered a site of failure after BCS and most importantly, this balanced the comparison between inverse- and forward-planned techniques.^6–8^ Inverse planned techniques like VMAT and IMRT, by virtue of their variable beam angles, can more effectively drive dose into the skin while the forward planned technique (FiF) cannot overcome the skin-sparing effect of the fixed oblique angles and so is invariably underdosed by FiF.^6,7^ This cropping therefore allows a more anatomically based, clinical comparison of breast target dosimetry.^8^

All organs at risk in all patients were delineated according to the RTOG 1005 protocol (NCT01349322). To minimise inter observer variation, one radiation oncologist performed delineation of all structures in all patients, on a single TPS (Varian Eclipse v13.5, Varian Medical Systems, USA). At least two of the participating radiation oncologists verified these contours before the treatment planning study.

We characterised the breast size as “small” or“large” based on breast volume (≤975 cc versus >975 cc), and characterised cardiac anatomy as “favourable” or “unfavourable” based on the cardiac contact distance.^9,10^

### Plan Quality Metric(PQM) scoring

The use of PQM as a relative scoring system is designed to remove ambiguity while providing directly comparative results for each dosimetric parameter and the overall plan in total.^11^

To be included in the PQM scoring schema, parameters had to be relevant to clinical outcomes and/or recommended for level two reporting by the International Commission on Radiation Units and Measurements (ICRU) Report 83.^12–15^ As a result of discussions between participating radiation oncologists using a nominal group technique, we identified a total of 53 candidate dosimetric parameters and achieved a consensus on parameter selection and scoring (details available in the Supplementary Material 1). The resulting PQM scoring schema was composed of 12 sub components, each having a unique metric quantity and value function (Table 1).

**Table 1.** Plan Quality Metric (PQM) scoring criteria (maximum possible score for any plan = 100)

Structure	Metric	Definition	PQM Score Range		
			Minimum (Metric Value)	Maximum (Metric Value)	Rationale for Inclusion
**PTV**	V_95%_ (%)	Volume receiving 95% of prescribed dose	0 (<90%)	10 (100%)	ICRU 83 level two reporting recommendation (15)
	D_95%_ (%)	Dose received by 95% of volume	0 (<90%)	10 (100%)	ICRU 83 level two reporting recommendation (15)
	D_2%_ (%)	Dose received by 2% of volume	0 (>110%)	5 (<105%)	ICRU 83 level two reporting recommendation (15)
	RTOG H.I.	Homogeneity Index (D_2%_ - D_98%_/ D_50%_)	0 (>0.2)	5 (<0.05)	ICRU 83 level two reporting recommendation (15)
	V_110%_ (cc)	Volume receiving 110% or more of prescribed dose	0 (>15 cc)	10 (0 cc)	Risk of skin toxicity and long term breast pain (12)
**Ipsilateral Lung**	D_mean_ (Gy)	Mean Dose	0 (>27 Gy)	10 (<7 Gy)	Risk of radiation induced pneumonitis (13)
	V_20_ (%)	Volume receiving 20 Gy	0 (>35%)	10 (<5%)	Risk of radiation induced pneumonitis (13)
**Heart**	D_mean_ (Gy)	Mean Dose	0 (>26 Gy)	10 (<4 Gy)	Risk of radiation induced heart disease, pericarditis (13)
	V_30_ (%)	Volume receiving 30 Gy	0 (>46%)	10 (<5%)	Risk of radiation induced pericarditis (13)
	V_25_ (%)	Volume receiving 25 Gy	0 (>10%)	10 (<1%)	Risk of radiation induced myocardial dysfunction (13)
**Right Breast**	V_4_ (%)	Volume receiving 4 Gy	0 (>10%)	5 (0%)	Risk of radiation induced carcinogenesis (14)
	D_mean_ (Gy)	Mean dose	0 (>5 Gy)	5 (<2 Gy)	Risk of radiation induced carcinogenesis (14)

### Treatment planning

Each PTV_Plan was prescribed 46 Gy in 2 Gy fractions with the following objectives:

1. V_95%_ > 95% for each respective PTV_Eval,
2. Heart D_mean_ < 26 Gy, and;
3. Left Lung V_20Gy_< 30%.

If the Heart or Lung criteria were not met, the PTV_Plan constraint was relaxed to V_95%_ > 90%. The PQM scoring schema was communicated *a priori* to the medical physicist undertaking planning. To minimise inter-planner variability, one medical physicist optimised plans for all patients on a single TPS (Varian Eclipse v13.5; AAA algorithm) and delivery platform (VarianTrueBeam v2.5; Millennium 120 MLC).

The planning process was constrained to resemble reasonable work practice and to control planning time bias.^11^ Once the minimum criteria were met, five further optimisation runs (over two days) were permitted to improve plan quality.^11^ The medical physicist defined the number of iterations in each optimisation run. Finally, the plans had to be deliverable within a 15 min time slot on the delivery platform. This was calculated by using each plan’s control point monitor units and interbeam transition time (IMRT) or gantry rotation speed (VMAT). Once the optimisation limit of five runs was reached, the plan with maximum PQM score was selected for analysis.

### Planning technique

FiF planning utilised two half-beam blocked tangential 6 MV beams (medial and lateral tangent with the gantry at 310^O^ and 140^O^) with source-to-surface distance (SSD) of 100 cm. The PTV was shaped in beams eye view (BEV) using an MLC with a margin of 5 mm for penumbra. Regions receiving more than 110% of prescribed dose were reduced with multiple subfields of medial and lateral tangents.^7^

IMRT planning utilised five tangential 6 MV static fields (gantry at 300^O^, 330^O^, 45^O^, 100^O^, and 150^O^) with SAD technique and inversely optimised with Dose Volume Optimization (DVO) algorithm.^7^

VMAT planning utilised two continuous 6 MV Hemi-arcs (starting angle 300^O^ and ending angle 150^O^; total 210^O^) with SAD technique and inversely optimised with Progressive Resolution Optimizer (PRO3) algorithm.^16^

### Statistical analysis

**Figure 1.**
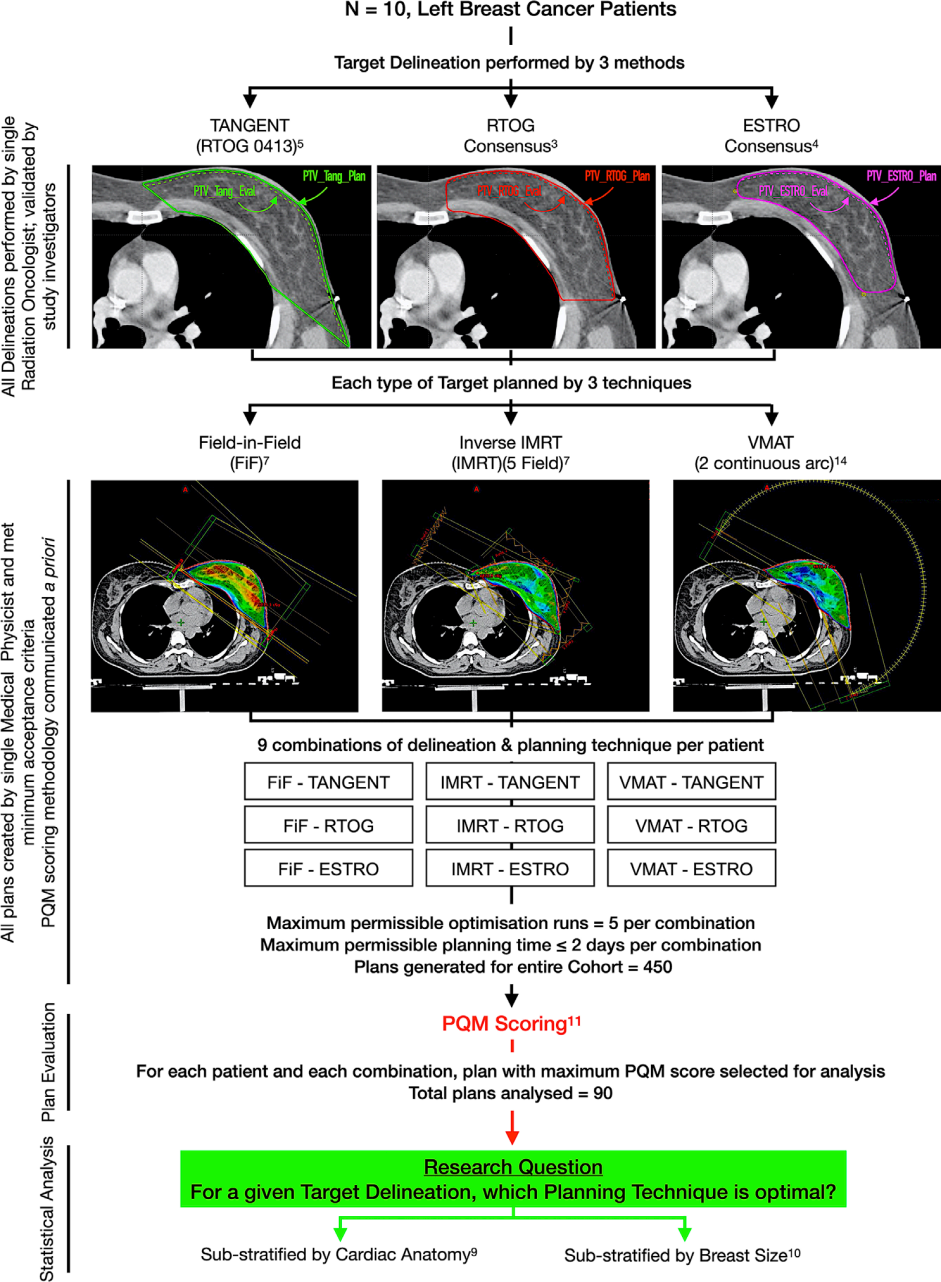
Study schema. *Abbreviations*: ESTRO, European SocieTy for Radiotherapy and Oncology; IMRT, Intensity Modulated Radiotherapy Technique; PQM, Plan Quality Metric; RTOG, Radiation Therapy & Oncology Group; VMAT, Volumetric Modulated Arc Therapy

The performance of each planning technique for each delineation protocol was compared using the PQM and dosimetric data obtained, as summarised in Figure 1.

Continuous variables were reported as mean ± standard deviation (SD), and categorical variables were reported as frequencies and percentages. The normality of continuous variables was tested with Shapiro–Wilk and Shapiro–Francia tests.

We used a two-way repeated measures analysis of variance (RM-ANOVA) to find significant associations, after correcting for any possible interaction between target delineation protocol and planning technique in each ANOVA model. A Bonferroni correction was applied to avoid the likelihood of incorrectly rejecting a null hypothesis. The significance level was set at <0.005 (0.05/9). All analyses were performed in Stata 14.2 (StataCorp, College Station, USA).

## Results

A total of 450 plans were generated for the entire cohort. Ninety plans were selected based on PQM score and minimum acceptance criteria. The results of dosimetric comparisons and all data associated with this analysis are presented in the Supplementary Material 1 and 2.

### PQM score comparison of planning technique based on delineation protocol (Figure 2, Table 2)

**Table 2.** PQM data for all combinations of planning technique and delineation protocols & results of RM-ANOVA for all comparisons (Significance level: p < 0.005). For sub-score PQM data of cardiac anatomy and breast size, please see Supplementary Material 1.

	PQM scores for the entire study cohort								
	FiF-ESTRO	IMRT-ESTRO	VMAT-ESTRO	FiF-RTOG	IMRT-RTOG	VMAT-RTOG	FiF-TANG	IMRT-TANG	VMAT-TANG
PQM Parameters	Mean(SD)	Mean(SD)	Mean (SD)	Mean (SD)	Mean (SD)	Mean (SD)	Mean (SD)	Mean (SD)	Mean (SD)
Total Score for PTV (Max 40)	22.90 (4.63)	32.60 (1.08)	28.83 (2.41)	13.03 (8.62)	31.46 (1.37)	25.96 (2.81)	18.13 (4.85)	31.86 (1.17)	27.00 (2.73)
Total Score for Left Lung (Max 20)	16.40 (2.17)	13.60 (2.50)	8.00 (3.02)	10.70 (2.75)	5.90 (1.79)	3.70 (1.70)	17.50 (1.90)	10.90 (2.64)	7.90 (2.92)
Total Score for Heart (Max 30)	23.70 (2.00)	22.70 (2.79)	18.20 (4.24)	14.10 (2.56)	17.30 (2.67)	14.70 (5.44)	22.70 (3.62)	20.10 (3.45)	17.30 (3.43)
Total Score for Right Breast (Max 10)	9.80 (0.48)	6.70 (1.55)	3.10 (0.91)	8.25 (1.77)	4.15 (1.62)	2.65 (0.88)	10.00 (0.00)	6.75 (1.48)	3.85 (1.65)
Combined Total Score (Max 100)	72.80 (5.27)	75.60 (5.89)	58.13 (7.57)	46.08 (12.80)	58.81 (4.10)	47.01 (7.25)	68.33 (6.52)	69.61 (5.54)	56.05 (5.43)

Results of RM-ANOVA stratified by delineation technique								
	ESTRO			RTOG			TANGENT		
PQM Parameters	IMRT *vs* FIF	VMAT *vs* FIF	VMAT *vs* IMRT	IMRT *vs* FIF	VMAT *vs* FIF	VMAT *vs* IMRT	IMRT *vs* FIF	VMAT *vs* FIF	VMAT *vs* IMRT
Total Score for PTV (Max 40)	<0.001	0.001	0.027 (ns)	<0.001	<0.001	0.002	<0.001	<0.001	0.005
Total Score for Left Lung (Max 20)	0.009 (ns)	<0.001	<0.001	<0.001	<0.001	0.039 (ns)	<0.001	<0.001	0.006 (ns)
Total Score for Heart (Max 30)	0.422 (ns)	<0.001	0.001	0.012 (ns)	0.629 (ns)	0.040 (ns)	0.040 (ns)	<0.001	0.027 (ns)
Total Score for Right Breast (Max 10)	<0.001	<0.001	<0.001	<0.001	<0.001	0.001	<0.001	<0.001	<0.001
Combined Total Score (Max 100)	0.304 (ns)	<0.001	<0.001	<0.001	0.731 (ns)	<0.001	0.637 (ns)	<0.001	<0.001

Results of RM-ANOVA stratified by delineation technique and sub stratified by breast size								
	ESTRO			RTOG			TANGENT		
PQM Parameters - Large Breast	IMRT *vs* FIF	VMAT *vs* FIF	VMAT *vs* IMRT	IMRT *vs* FIF	VMAT *vs* FIF	VMAT *vs* IMRT	IMRT *vs* FIF	VMAT *vs* FIF	VMAT *vs* IMRT
Combined Total Score (Max 100)	0.426 (ns)	<0.001	<0.001	<0.001	0.680 (ns)	<0.001	0.806 (ns)	<0.001	<0.001
PQM Parameters - Small Breast									
Combined Total Score (Max 100)	0.545 (ns)	0.043 (ns)	0.012 (ns)	0.099 (ns)	0.943 (ns)	0.113 (ns)	0.700 (ns)	0.067 (ns)	0.031 (ns)

Results of RM-ANOVA stratified by delineation technique and sub stratified by cardiac anatomy								
	ESTRO			RTOG			TANGENT		
PQM Parameters - Favourable	IMRT *vs* FIF	VMAT *vs* FIF	VMAT *vs* IMRT	IMRT *vs* FIF	VMAT *vs* FIF	VMAT *vs* IMRT	IMRT *vs* FIF	VMAT *vs* FIF	VMAT *vs* IMRT
Combined Total Score (Max 100)	0.429 (ns)	<0.001	<0.001	<0.001	0.163 (ns)	0.001	0.737 (ns)	0.001	<0.001
PQM Parameters - Unfavourable									
Combined Total Score (Max 100)	0.582 (ns)	0.008 (ns)	0.002	0.350 (ns)	0.110 (ns)	0.019 (ns)	0.722 (ns)	0.012 (ns)	0.006 (ns)

**Figure 2.**
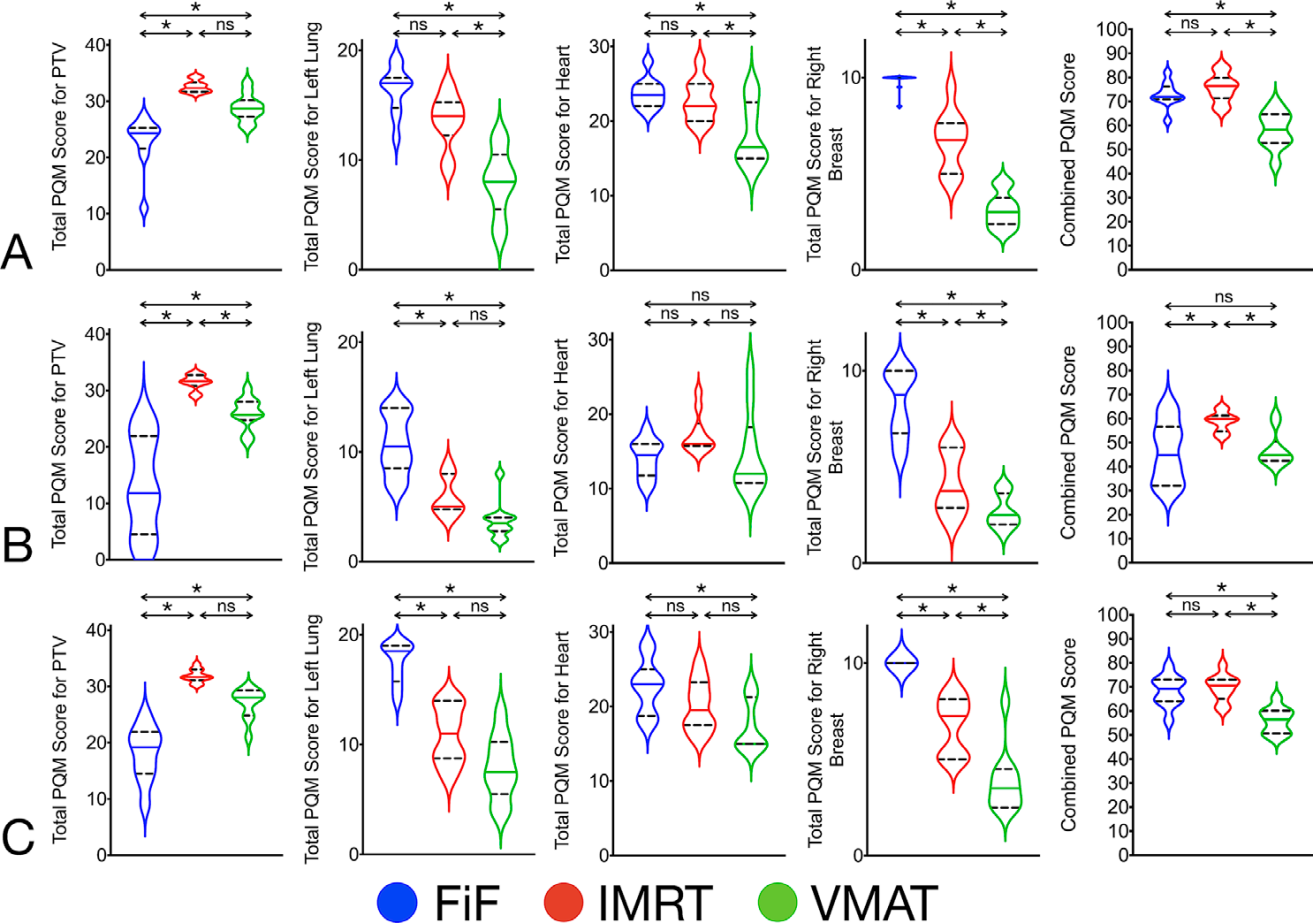
Violin plots for PQM score comparison of planning techniques (FiF, IMRT, VMAT) based on delineation protocol. Significant comparisons are marked with an asterisk (*). (A) Results for ESTRO delineation. (B) Results for RTOG delineation. (C) Results for RTOG 0413 (Tangent) WBI Protocol. Abbreviations: ESTRO, European SocieTy for Radiotherapy and Oncology; FiF, Field-in-field IMRT; IMRT, Intensity Modulated Radiotherapy Technique; ns, not significant; PQM, Plan Quality Metric; RTOG, Radiation Therapy & Oncology Group; VMAT, Volumetric Modulated Arc Therapy

1. The ESTRO Consensus Guideline (Figure 2A)
  For plans based on the ESTRO guideline, the combined PQM scores for each planning technique demonstrated that FiF was comparable to IMRT (FiF *vs* IMRT, *p* = 0.304) and that both achieved higher scores than VMAT (FiF *vs* VMAT, *p* < 0.001; IMRT *vs* VMAT, *p* < 0.001).
  Analysis of individual subscores showed that the PTV_ESTRO_Eval score was the least for FiF (FiF *vs* VMAT, *p* = 0.001; FiF *vs* IMRT, *p* < 0.001) and that scores for VMAT and IMRT were comparable (VMAT *vs* IMRT, *p* = 0.027). Individual subscores for left lung, heart, and right breast were lower for VMAT compared to both FiF and IMRT.
2. The RTOG Consensus Guideline
  For plans based on the RTOG guideline, the combined PQM scores were higher for IMRT than both FiF and VMAT (IMRT *vs* FiF, *p* < 0.001; IMRT *vs* VMAT, *p* < 0.001), while the score for FiF was comparable to VMAT (FiF *vs* VMAT, *p* = 0.731).
  Analysis of individual subscores showed that the PTV_RTOG_Eval score was the highest for IMRT (IMRT *vs* FiF, *p* < 0.001; IMRT *vs* VMAT, *p* = 0.002) and that VMAT achieved a higher score than FiF (VMAT *vs* FiF, *p* < 0.001). Individual subscores for left lung and right breast were higher for FiF than both IMRT and VMAT. All three techniques scored comparably for heart subscores.
3. RTOG 0413 (Tangent) WBI Protocol (Figure 2C)
  For plans based on the Tangential delineation, the combined PQM scores were comparable between FiF and IMRT (FiF *vs* IMRT, *p* = 0.637) and both were higher than VMAT (FiF *vs* VMAT, *p* < 0.001; IMRT *vs* VMAT, *p* < 0.001).
  Analysis of individual subscores showed that the PTV_Tang_Eval score was the highest for IMRT (IMRT *vs* FiF, *p* < 0.001; IMRT *vs* VMAT, *p* = 0.005) and that VMAT achieved a higher score than FiF (VMAT *vs* FiF, *p* < 0.001). Individual subscores for left lung and right breast were higher for FiF compared to both IMRT and VMAT. Subscore for the heart were higher for FiF compared to VMAT (FiF *vs* VMAT, *p* < 0.001), while other comparisons were not significantly different (FiF *vs* IMRT, *p* = 0.040; IMRT *vs* VMAT, *p* = 0.027).

### PQM score comparison of planning techniques stratified by breast size and cardiac anatomy (Figure 3, Table 2)

**Figure 3.**
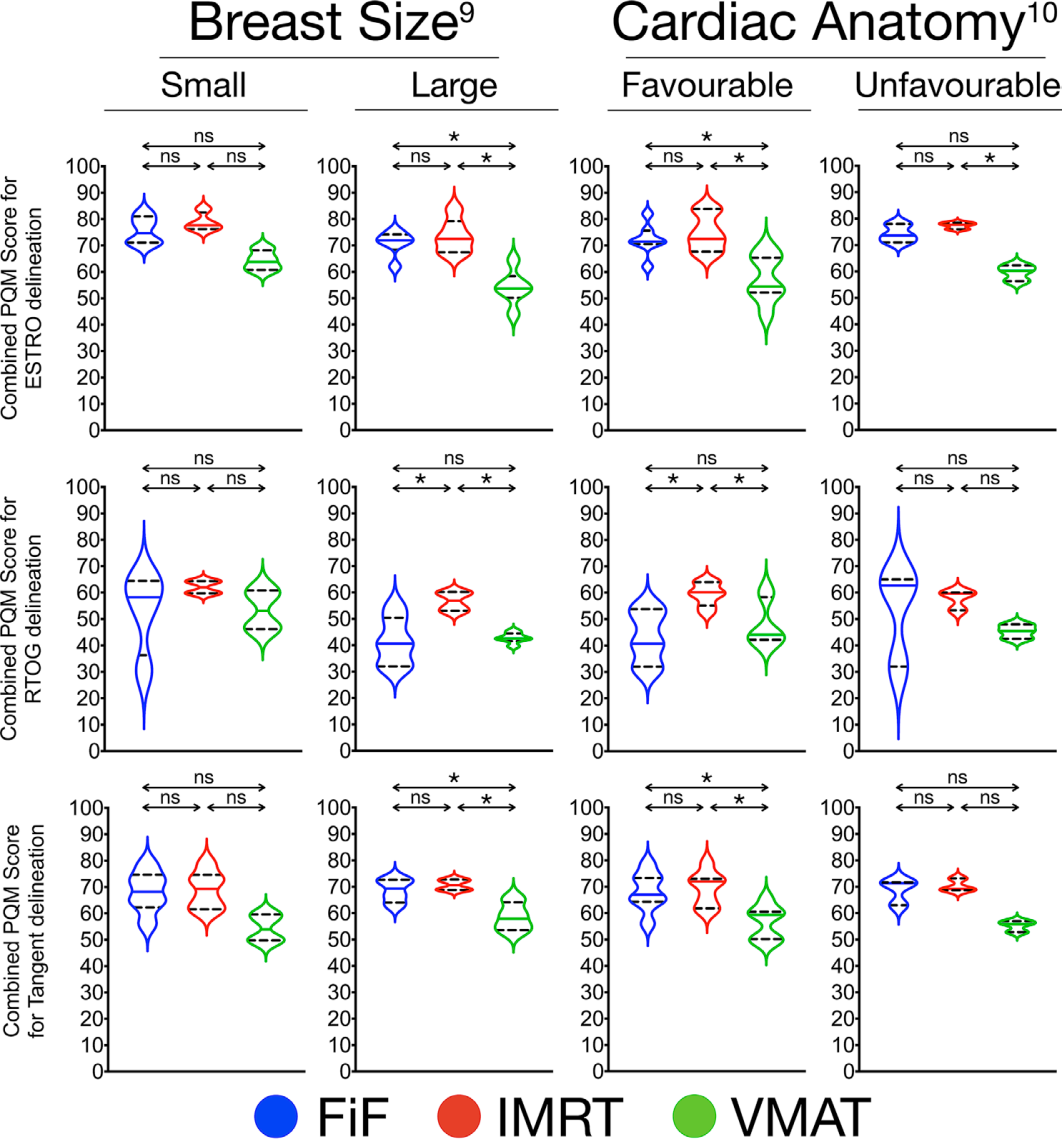
Violin plots for PQM score comparison of planning techniques (FiF, IMRT, VMAT) based on delineation protocol, sub stratified by cardiac anatomy and breast size.Significant comparisons are marked with an asterisk (*).Abbreviations: ESTRO, European SocieTy for Radiotherapy and Oncology; FiF, Field-in-field IMRT; IMRT, Intensity Modulated Radiotherapy Technique; ns, not significant; PQM, Plan Quality Metric; RTOG, Radiation Therapy & Oncology Group; VMAT, Volumetric Modulated Arc Therapy

1. Breast Size
  Analysis of combined PQM scores demonstrated that in patients with small breasts (*N* = 4), all planning techniques achieved comparable scores, irrespective of delineation protocol.
  In patients with large breasts (*N* = 6) contoured using ESTRO guideline and RTOG 0413 (Tangent) WBI protocol, FiF and IMRT achieved higher scores than VMAT. However using the RTOG guideline, combined PQM scores for IMRT were higher than FiF and VMAT, with the scores for FiF and VMAT being comparable.
2. Cardiac Anatomy
  Analysis of combined PQM scores demonstrated that in patients with unfavourable anatomy (*N* = 3), all planning techniques achieved comparable scores, irrespective of delineation protocol. The exception was the comparison between IMRT and VMAT for the ESTRO guideline, in which IMRT achieved a significantly higher combined PQM score.
  In patients with favourable cardiac anatomy (*N* = 7), using the ESTRO guideline and RTOG 0413 (Tangent) WBI protocol, FiF and IMRT achieved higher combined PQM scores than VMAT. When using the RTOG guideline, the combined PQM scores for IMRT were higher than both FiF and VMAT.

Subscore PQM data and *p*-values for both RM-ANOVA analyses are shown in the Supplementary Material 1 and 2.

## Discussion

We believe that this study is the first to formally analyse the interplay between treatment planning technique and breast delineation protocol. Our analysis of total PQM scores found that for the ESTRO guideline and RTOG 0413 (Tangent) WBI protocol, FiF and IMRT were comparable, and both scored higher than VMAT. However, on analysing the RTOG guideline, IMRT scored higher than both FiF and VMAT with the scores for FiF and VMAT being comparable. These results were also applicable to patients with large-sized breasts or favourable cardiac anatomy.

These results are not unexpected as each planning technique sacrifices performance in one facet to achieve a gain in another. The underperformance of VMAT planning is explained by the inherent trade-off between better target coverage at the cost of higher OAR doses.^16^ The equivalence of FiF and IMRT planning can be explained by the higher OAR sparing (but with lower target coverage) resulting in a combined PQM score which was comparable to IMRT (higher target coverage but lower OAR sparing).

An analysis similar to the present study investigated hypo-fractionated radiotherapy delivery techniques with sequential or simultaneous integrated boost utilising a combination of 3DCRT, IMRT or VMAT. Delineation was performed utilising the ESTRO guideline, and PQM scoring was based on the protocol compliance criteria of RTOG 1005 (NCT01349322). The authors reported similar conclusions in which PQM scores for VMAT were significantly less than IMRT or 3DCRT.^17^ Another dosimetric comparison between conventionally fractionated radiotherapy delivery techniques [FiF, tangential IMRT (tIMRT) and VMAT (tangential & continuous)] utilising the RTOG guideline reported contrasting results. The authors concluded that both VMAT techniques achieved better dosimetric results when compared to tIMRT and FiF techniques.^18^ The results of both analyses highlight the interplay of planning techniques with delineation protocols and strengthen the central premise of our study, which is to disentangle the influence of delineation protocol on planning techniques.

As techniques and delineation guidelines evolve, individual department preferences will converge on one technique and delineation method. Consequently, the question we investigated was empirically developed from the perspective of the radiation oncologist (what is the optimal planning technique for the type of target delineation performed?). This prompted a discussion about the most informative method for statistical analysis and the choice of a relative comparison method (PQM scores). Rather than undertaking a comparison of absolute superiority based on dosimetric criteria alone for a preferred planning technique and delineation protocol, we sought to comprehend the contributing factors leading to better combinations of available techniques and delineation protocols. Our analysis demonstrates that the selection of planning technique and delineation method has significant co-dependence.

The literature on mathematical DVH reduction tools to compare different treatment plans is abundant but varies in complexity from simple to involved. On one end of the spectrum is the use of a binary scoring system based on a defined set of objectives/constraints and on the other, an intricate summation of objectives which are scored conditionally utilising exponential functions.^19–21^ While an argument can be made for both approaches, the adoption rate of these DVH reduction tools will ultimately be judged by the radiation oncologist, which may also vary between different countries. In the UK, the tasks handled by Clinical Oncologists often extend beyond radiotherapy alone whereas in other countries, Radiation Oncologists are focused on radiotherapy alone.^22^ The PQM scoring method offers simplicity without sacrificing granularity, and its robustness has been assessed by changing the weights of subscores, which did not change the order of the planning techniques.^11,17^

Besides the modest number of patients included in our analysis, the relative weights assigned to the PQM scoring schema can also be criticised. The scoring mechanism does not seek to define the best plan; instead, it objectively scores each plan based on the *a priori* departmental priorities which have been established before any planning takes place.^9^ A different weighting of scores for alternative clinical priorities could produce different results, although we believe that our weightings are based on realistic and clinically relevant objectives.

It is important to emphasise that the results of our analysis are highly dependent on the TPS platform we used and the planning proficiency of our medical physicist. An inter-institutional analysis incorporating more planners with variable proficiency and a variety of TPS platforms based on a common imaging dataset would result in broader, more generalisable conclusions.

The decision to limit the maximum permissible optimisation runs along with a time limit to perform them in, could be criticised as restrictive. However, these restrictions served as a control for the bias associated with cumulative planning time and also imposed a real-world constraint evident in any high-volume centre striving to achieve the appropriate balance between planning time, plan complexity and practical deliverability.^9^ In contrast, given the ideal scenario of indefinite time and iterations, a Pareto-optimal planning strategy could be achieved by producing an enormous number of plans and creating multiple Pareto-optimal fronts for each scored parameter, but analysing and comprehending the optimal solution for multiple parameters in a two-dimensional space with such an approach would be challenging.^23^

Several questions have not been addressed, most importantly the influence of voluntary Deep Inspiration Breath Hold and inclusion of regional nodal irradiation on our results.^7^ Our group will address these avenues of research in future analyses.
